# Interaction between IGF-IR and ER Induced by E2 and IGF-I

**DOI:** 10.1371/journal.pone.0062642

**Published:** 2013-05-21

**Authors:** Zhenghong Yu, Weimin Gao, Enze Jiang, Fang Lu, Luo Zhang, Zhaorong Shi, Xinxing Wang, Longbang Chen, Tangfeng Lv

**Affiliations:** 1 Department of Medical Oncology, Jinling Hospital, Nanjing, China; 2 Department of Environmental Toxicology, The Institute of Environmental and Human Health, Texas Tech University, Lubbock, Texas, United States of America; 3 Nanjing University School of Medicine, Nanjing, China; 4 Nanjing Foreign Language School, Nanjing, China; 5 Hospital office, Jinling Hospital, Nanjing, China; 6 Department of Respiratory Medicine, Jinling Hospital, Nanjing, China; Karolinska Institutet, Sweden

## Abstract

Estrogen receptor (ER) is a nuclear receptor and the insulin-like growth factor-I (IGF-I) receptor (IGF-IR) is a transmembrane tyrosine kinase receptor. Estrogen and IGF-I are known to have synergistic effects on the growth of breast cancer cells. Recently, non-nuclear effects of ER have been under investigation. To study the mechanism involved in this process, we have used MCF-7 breast cancer cell lines transfected with IGF-IR anti-sense cDNA (SX13, MCF-7^SX13^) that resulted in 50% reduction of IGF-IR. In MCF-7 cells, estradiol (E2) and IGF-I induced the rapid association of ER to IGF-IR, however, the interaction was abrogated in MCF-7^SX13^ cells. In addition, NWTB3 cells (NIH3T3 cells overexpressing IGF-IR) were transiently transfected with ERα, the ER-IGF-IR interaction was induced by both E2 and IGF-I. Moreover, ERα regulated the IGF-I signaling pathways through phosphorylation of ERK1/2 and Akt and the interaction of ER-IGF-IR potentiated the cell growth. Finally, E2 and IGF-I stimulated translocation of ER from the nucleus to the cytoplasm. Taken together, these findings reveal that the interaction of the ER and IGF-IR is important for the non-genomic effects of ER.

## Introduction

All tissues and cells respond simultaneously to multiple growth and differentiation factors that influence their development, growth, and differentiation. Many of these factors are extracellular signaling molecules that reach the cells *via* the circulation or from local paracrine sources. To influence the biological responses of the cells, these factors or ligands must interact with receptors that then signal the intracellular events, culminating in a biological response. Some receptors are expressed on the surface of the cells, including the receptor tyrosine kinase family [1], integrins [2], and the serpentine receptors [3]. Other receptors are found intracellularly either in the cytoplasm or the nucleus, such as the nuclear receptors for steroid hormones [4]. Since cells respond to multiple signaling molecules simultaneously, it has recently become of major interest to examine the responses of various cells to receptor activation from multiple classes, rather than studying a single ligand-receptor response in isolation [5].

Given the effects of steroid hormones and growth factors on the proliferation of cancer cells, the signaling cross-talk between the tyrosine kinase receptors and the nuclear receptors has become a particularly important area of research. Tissues including breast [6], uterine [7] and endometrial cancers [8] are responsive to both estradiol (E2) and insulin-like growth factors (IGF). There are a number of cell lines that have been proven useful in these investigations, such as the MCF-7 breast cancer cell line that expresses both estrogen receptor (ER) and insulin-like growth factor-I (IGF-I) receptor (IGF-IR). These cells have been shown to respond to these ligands with increased levels of cellular proliferation, enhanced signaling events, as well as expression of cell cycle-related molecules [9]. Interestingly, the activated IGF-IR and ER demonstrate additive or synergistic effects when both ligands are administered simultaneously, strongly indicated cross-talk between these receptors from different structural families [10].

IGF-IR is a tyrosine kinase receptor that interacts with its ligand *via* the extracellular domain and then leads to a conformational change in the receptor, which undergoes autophosphorylation on tyrosine residues [1]. A number of intracellular protein substrates interact with the receptor then undergo tyrosine phosphorylation, leading to several major signaling cascades. For instance, the PI3 kinase pathway can be activated by the insulin-receptor substrate (IRS), a major substrate of the IGF-IR. This activation leads to further phosphorylation and activation of PKB/Akt kinase. Another important substrate is Shc which binds Grb2/mSOS and eventuates in the activation of the Ras/Raf/MAP kinase pathway [11]. Additionally, MAP kinase (MAPK) pathways are also involved in IGF-IR signaling [12].

The ERs are nuclear receptors, *i.e*. they are bound by ligand (estradiol) and bind the promoter regions of various genes, thereby affecting transcription of these genes [13]. Of particular interest is the recent finding that ER activation by ligand binding results in events that suggest a non-genomic effect, *i.e*. the effect is very rapid and not related to gene expression [14]. Investigators have also studied synergism between the two receptors (IGF-IR and ER) and have demonstrated that estrogen can affect the IGF system [15], [16]. IGF-IR activation increases ER-mediated enhancement of transcriptional activity *via* a number of pathways including Erk 1/2 [17], Akt [18], pp90rsk1 [19], pp90rsk2 [20], or JNK [21]. Furthermore, it has been suggested that ER could rapidly bind to IGF-IR and result in MAPK activation, which in turn leads to ER activation in the nucleus, presumably through translocation of ER in the cellular components [22].

The objectives of the present study were to not only further confirm the interactions between ER and IGF-IR, but to determine their consequential biological significance. We have performed experiments in two different cell lines, including MCF-7 breast cancer cell line that expresses both ER and IGF-IR and NIH3T3 fibroblast cell line with undetectable endogenous ER. Our results demonstrate presence of physical interaction of these two receptors and their biological importance.

## Materials and Methods

### Chemicals and antibodies

Recombinant human IGF-I was obtained from Genentech (San Francisco, CA). Recombinant human 17β-estradiol (E2), phenylmethylsulfonyl (PMSF), leupeptin, aprotinin, and protein G-Sepharose were obtained from Sigma Chemicals (St Louis, MO). ICI 182780 was purchased from Sigma. Triton X-100, sodium dodecyl sulfate (SDS), and nitrocellulose membranes were obtained from Bio-Rad laboratories (Richmond, CA). Rabbit polyclonal antibodies to ERα (HC20) and the IGF-I receptor beta subunit (C20) were purchased from Santa Cruz Biotechnology (Santa Cruz, CA). Monoclonal anti-Actin (Clone AC) was obtained from Sigma. p44/42 MAPK (ERK1/2), phospho-ERK1/2 (Thr202/Tyr204), and Akt (11E7), phospho-Akt (Ser473) antibodies were purchased from Cell Signaling (Danvers, MA). Horseradish peroxidase-conjugated anti-rabbit and anti-mouse immunoglobulins were purchased from Amersham Corp. (Arlington Heights, IL). Electrochemiluminescence (ECL) kit was obtained from NEN Life Science Products (Boston, MA). The CyQUANT Cell Proliferation kit was purchased from Molecular Probes (Eugene, OR). Cell culture media and reagents were purchased from Biofluids Inc. (Rockville, MD).

### Cell culture

MCF-7 cells from ATCC (Rockville, MD) were maintained in Improved Minimum Essential Medium (IMEM) supplemented with 10% fetal bovine serum (FBS). MCF-7 stably transfected cells were maintained in IMEM containing 10% FBS supplemented with 800 µg/mL G418 (Geneticin, GIBCO, Grand Island, MD). NIH3T3 cells were maintained in Dulbecco's Modified Eagle's Medium (DMEM) supplemented with 10% FBS. NIH3T3 stably transfected cells were maintained in DMEM containing 10% FBS and 500 µg/mL G418. For cross-talk studies, the medium was changed to phenol red-free medium with the anti-estrogen ICI 182780 (10 nM) for 48 h to arrest cells in the G0/G1 phase. All medium used contained glutamine (2 mM), penicillin (100 IU/mL) and streptomycin (100 g/mL).

### Generation of MCF-7^SX13^, MCF-7^Neo^, and NWTB3 cells

MCF-7^SX13^ cells were generated from MCF-7 cells with IGF-IR knocking down by stably transfecting with an antisense IGF-IR cDNA (SX13). MCF-7^Neo^ cells were MCF-7 cells transfected with the same vector without the antisense construct (control). NWTB3 cells were NIH3T3 cells stably overexpression of IGF-IR with G418 selection.

### Cell proliferation assay

Cells were plated in 96-well plates (3000 cells/well) overnight in culture medium as described under cell culture and then transfected with human ER cDNA or empty vector pcDNA1 for 24 h. Transfected cells were synchronized in phenol-free DMEM supplemented with 0.1% BSA overnight and then stimulated with 10 nM IGF-I. CyQUANT cell proliferation assay was used to determine the density of cells following the manufacture's protocol. Fluorescence was measured using an HTS 7000 BioAssay reader (PerkinElmer Life Sciences, Boston, MA) with filters for 480 nm excitation and 520 nm emission.

### Immunoprecipitation

Cell lysates were prepared in lysis buffer [10 mM Tris, (pH 7.4), 150 mM NaCl, 1 mM EDTA, 1 mM EGTA, 0.5% Nonidet P40] containing protease inhibitors (2 mM PMSF, 10 mg leupeptin/ml, 10 mg aprotinin/ml) and phosphatase inhibitors (100 mM sodium fluoride, 10 mM sodium pyrophosphate, 2 mM sodium orthovanadate). Lysates were centrifuged at 12,000×g for 20 min at 4°C. Protein concentrations were determined using the BCA protein assay. Supernatants (250 μg of protein) were incubated with the respective antibodies at 4°C overnight. The immunocomplexes were precipitated with protein G-Sepharose for 1 h at 4°C. After two sequential washes using lysis buffer at a 1/2 dilution, the resulting pellets were boiled for 4 min in reducing Laemmli buffer containing 80 mM dithiothreitol. Sepharose beads were pelleted by centrifugation in a microcentrifuge at 12,000×g for 5 min. The supernatant was subsequently analyzed by SDS-polyacrylamide gel electrophoresis and then immunoblotting as described below.

### Immunoblotting

Cellular lysates (30 µg/sample) were resolved by SDS-gel electrophoresis in 8% polyacrylamide gels (for analysis of IGF-IR) or 10% polyacrylamide gels (for analysis of ERα) and transferred to nitrocellulose membranes. Membranes were blocked and incubated with the different antibodies. After extensive washings, immune complexes were detected with correspondent horseradish peroxidase conjugated with specific secondary antibody, followed by enhanced chemiluminescence reaction. Densitometry was performed by scanning the radiographs and then analyzing the bands with the MacBas V2.52 software (Fuji PhotoFilm).

### GFP assay

ERα was inserted into an EGFP-expression vector (Clontech, Palo Alto, California). NWTB3 cells were then transiently transfected with this vector using FuGENE (Roche, Basel, Switzerland) according to the manufacturer's instructions. After transfection, cells were treated with 10 nM E2, 10 nM IGF-I, or both for 20 min. The cells were harvested, centrifuged, and washed with PBS. The cell pellet was then resuspended in PBS. The GFP fluorescent expression was observed under a fluorescent microscope (LX71, Olympus, Japan). The differences among the groups were compared.

### Statistical analyses

The differences between two samples were evaluated by Student's *t*-tests. ANOVA was performed to test the differences among the groups. Differences were considered to be statistically significant at a *p*-value of less than 0.05. All of the *p*-values presented in this study are two-sided. The data were analyzed with SPSS13.0 statistical software.

## Results

### Association of IGF-IR to ERα is enhanced by IGF-I in MCF-7

**Figure 1:**
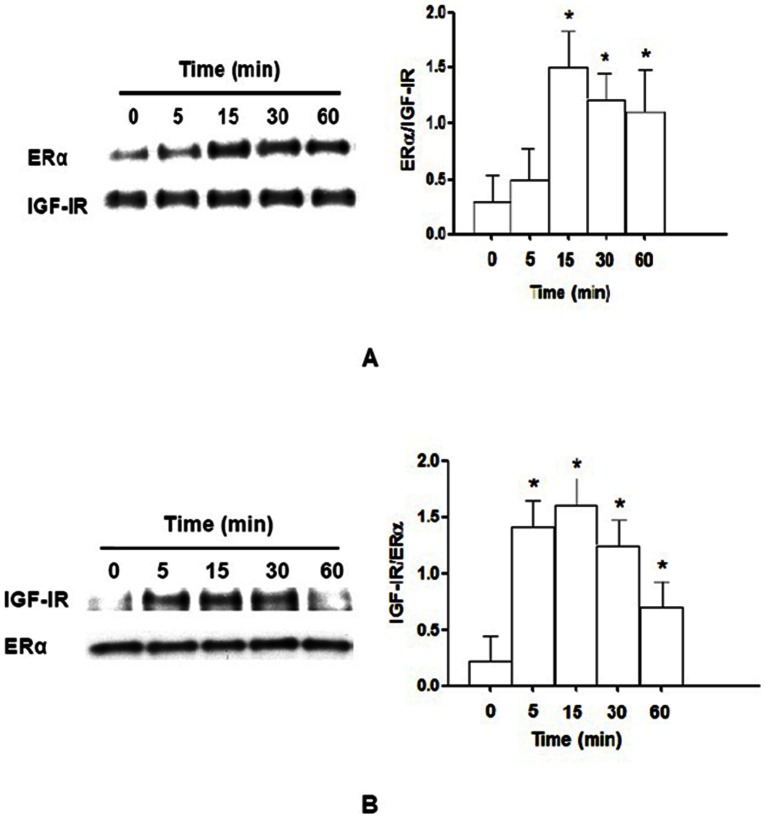
Binding of IGF-IR to ERα is stimulated by IGF-I in MCF-7 cells. MCF-7 cells were stimulated with 10 nM IGF-I for 0, 5, 15, 30, or 60 min. Protein lysates were subjected to immunoprecipitation with IGF-IR antibody and subsequently immunoblotted with ERα antibody (**A**) or immunoprecipitation with ERα antibody and subsequently immunoblotted with IGF-IR antibody (**B**). The ratio of ERα and IGF-IR rapidly increased from 0 to 15 min, and reached peak at 15 min and gradually decreased from 30 to 60 min (A). Also, the ratio of IGF-IR and ERα rapidly increased from 0 to 15 min, and reached peak at 15 min and gradually decreased from 30 to 60 min (B). *N* = 3, *, *P*<0.05 as compared to 0 min.

We performed a series of co-immunoprecipitation experiments of IGF-IRs and ER to elucidate the underlying mechanisms. MCF-7 cells expressing endogenous ER were stimulated with 10 nM IGF-I for 0–60 min (Figure 1). Cellular lysates were either immunoprecipitated with IGF-IR antibody and blotted with ERα antibody (Figure 1A) or immunoprecipitated with ERα antibody and blotted with IGF-IR antibody (Figure 1B). Similar patterns were observed after immunoprecipitation with either IGF-IR antibody or ERα antibody. Enhanced co-immunoprecipitation was detected as short as 5 min after IGF-I treatment and the levels of co-immunoprecipitation were time dependent (*P*<0.05, Figures 1A & 1B). The maximum effect was obtained within 15 min of IGF-I treatment (Figures 1A & 1B).

### IGF-I fails to enhance the association of IGF-IR and ERα in MCF-7^SX13^ cells

**Figure 2:**
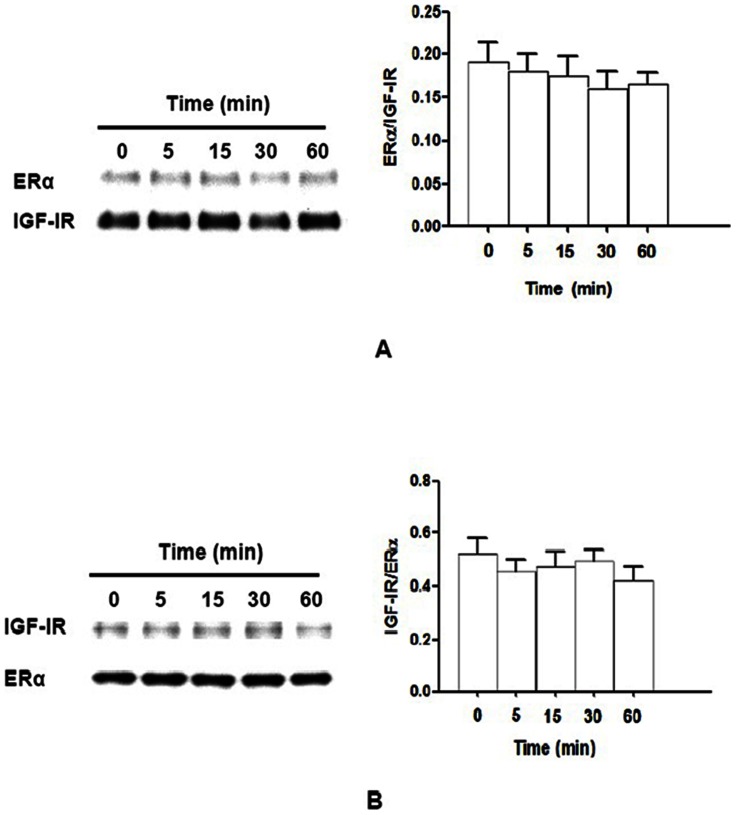
Binding IGF-IR to ERα is abrogated by IGF-I in MCF-7^SX13^ cells. MCF-7^SX13^ cells were stimulated with 10 nM IGF-I for 0–60 min. Protein lysates were subjected to immunoprecipitation with IGF-IR (A) or ERα (B) antibodies with subsequent immunoblotting of the precipitated fraction with ERα antibody (**A**) or IGF-IR antibody (**B**), respectively. The ratios of ERα/IGF-IR and IGF-IR/ERα at different time points were similar to each other, *N* = 3, *P*>0.05.

To establish function of the IGF-IR in ER-IGF-IR association, we used MCF-7^SX13^ cells. MCF-7^SX13^ cells have a 50% reduction of IGF-IR on western blotting analysis (data not shown). These cells were also stimulated with 10 nM IGF-I for 0–60 min. Cellular lysates were subjected to immunoprecipitation with IGF-IR antibody and blotted with ERα antibody (Figure 2A) or immunoprecipitated with ERα antibody and blotted with IGF-IR antibody (Figure 2B). Different from the effects observed in MCF-7^Neo^ cells (similar to MCF-7, data not shown), enhanced co-immunoprecipitation was not seen in MCF-7^SX13^ cells after IGF-I stimulation (Figures 2A & 2B).

### Association of IGF-IR to ERα is enhanced by IGF-I or E2 in NWTB3 cells

**Figure 3:**
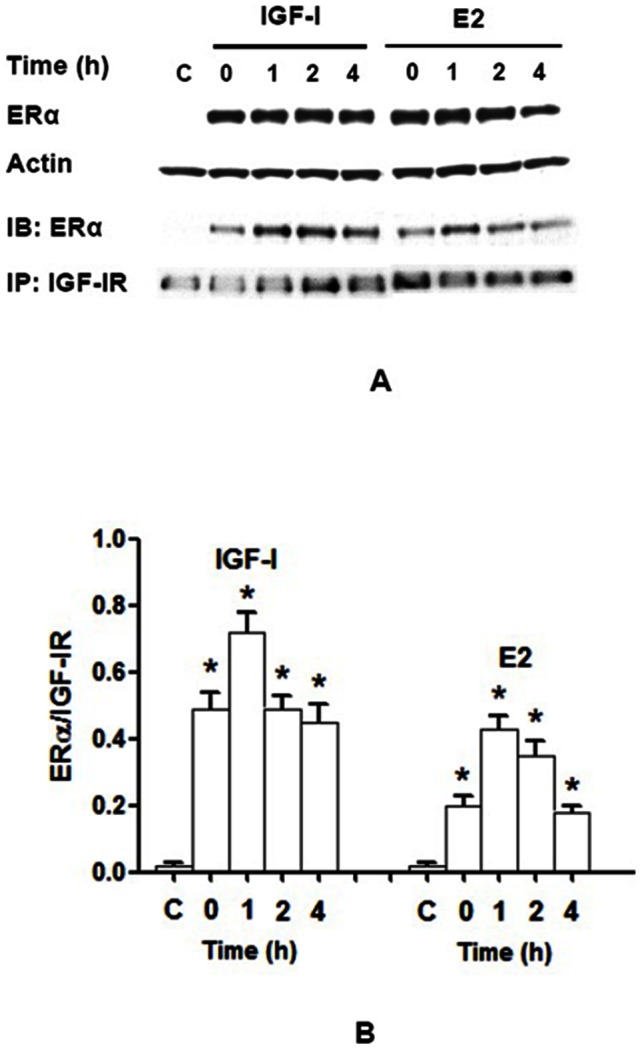
Binding of IGF-IR to ERα is stimulated by IGF-I or E2 in NWTB3 cells. NWTB3 cells transiently transfected with ERα were stimulated with 10 nM IGF-I or 10 nM E2 for 0–4 h. (**A**) Protein lysates were subjected to either immunoblotting with ERα antibody for determination of the expression of ERα or immunoprecipitated with IGF-IR antibody with subsequent immunoblotting of the precipitated fraction with ERα antibody. (**B**) The ratio of ERα/IGF-IR rapidly increased from 0 to 4 h, reached peak at 1 h after IGF-I stimulation, meanwhile, E2 treatment also enhanced the ratio with the peak at 1 h. C, NIH3T3 cells without ERα cDNA transfection. *N* = 3, *, *P*<0.05 as compared to the control groups.

To further examine the association between ERα and IGF-IR and the functional consequence, we transiently transfected NWTB3 cells (overexpressing IGF-IRs but were devoid of ER) with an exogenous ERα cDNA or vector control for 24 h. The cells were then stimulated with 10 nM IGF-I or 10 nM E2 for 1–4 h (Figure 3). Cellular lysates were subjected to immunoprecipitation with IGF-IR antibody and blotted with ERα antibody (Figure 3A). The maximum co-immunoprecipitation was detected within 1 h in NWTB3 cells after IGF-I or E2 stimulation (Figures 3A & 3B).

### Effect of ER on IGF-IR signaling pathways following IGF-I stimulation

**Figure 4:**
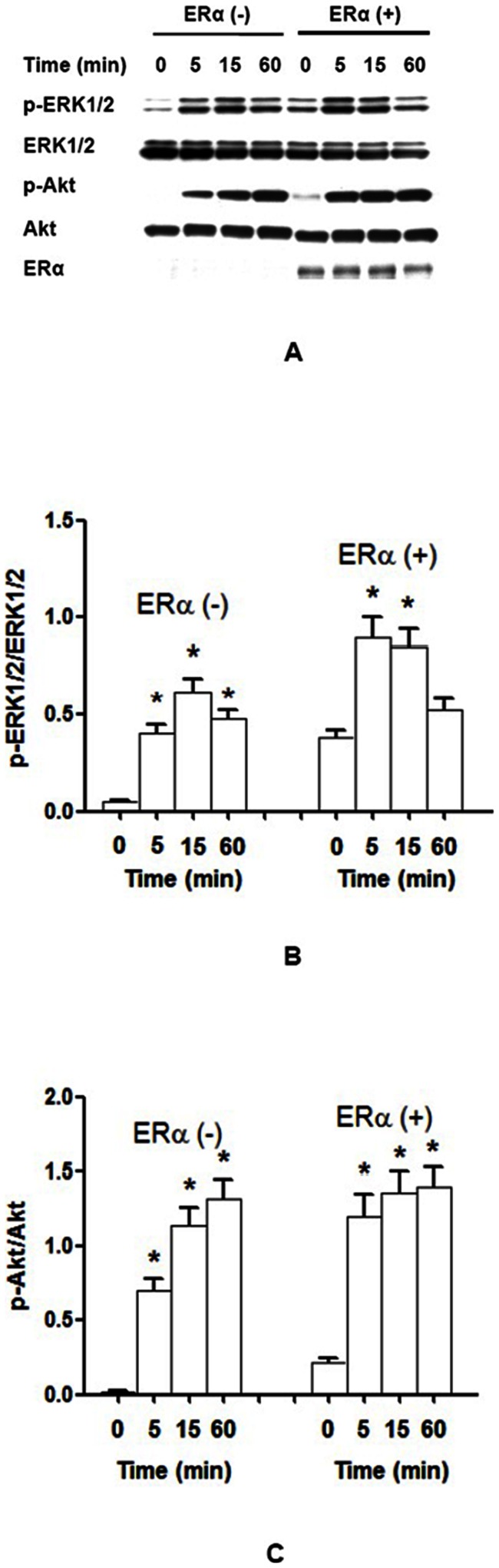
ERα potentiates the basal and maximum phosphorylation of ERK1/2 and Akt by IGF-I stimulation. NWTB3 cells transfected with ERα or vector control were stimulated with 10 nM IGF-I for 0–60 min. (**A**) Protein lysates were immunoblotted with phospho-ERK1/2, ERK1/2, phospho-Akt, Akt, or ERα antibodies. Transfection of ERα gradually increased phosphorylation of ERK1/2 and Akt after IGF-I stimulation. (**B**) The ratios of p-ERK1/2 *vs*. ERK1/2. The maximum phospho-ERK1/2 appeared in 5 min in ERα transfected NWTB3 cells after IGF-I stimulation, while it was 15 min in ERα-negative control cells. (**C**) The ratios of p-Akt *vs*. Akt. The maximum of phosphorylation of Akt was at 15 min in ERα-positive cells, but at 60 min in ERα-negative cells. *N* = 3, *, *P*<0.05 as compared to controls.

NWTB3 cells that were transiently transfected with ERα cDNA or vector control for 24 h and then stimulated with 10 nM IGF-I for 0–60 min. Cells with ERα cDNA transfection had higher levels of phosphorylation in both ERK1/2 and Akt as compared to controls (P<0.05, Figure 4A). The maximum phospho-ERK1/2 appeared in 5 min in ERα transfected NWTB3 cells after IGF-I stimulation, which was earlier than that seen in ERα-negative control cells (15 min) (Figures 4A & 4B). The maximum phospho-ERK1/2 in ERα transfected NWTB3 cells was 1.5-fold increased compared to ERα-negative control. The maximum of phosphorylation of Akt was at 15 min in ERα-positive cells, but at 60 min in ERα-negative cells (Figure 4A & 4C). Moreover, the phosphorylation of Akt reached 70% of maximum in ER-positive cells compared with only 50% of maximum in ER-negative control cells after 5 min of IGF-I stimulation (Figures 4A & 4C).

### Cell proliferation is enhanced in ERα-transfected NWTB3 cells by IGF-I stimulation

**Figure 5:**
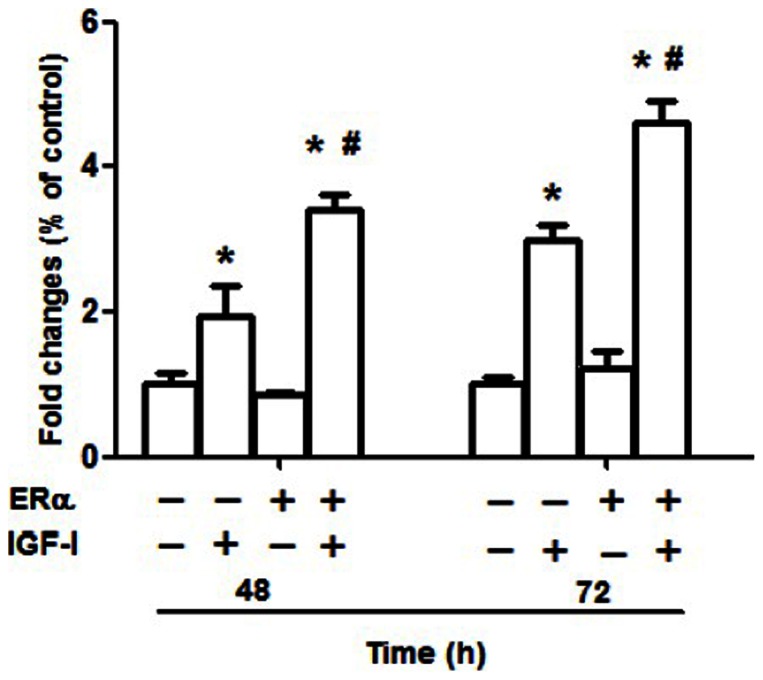
Cell proliferation is enhanced in ERα-transfected NWTB3 cells by IGF-I stimulation. To investigate the potential cross-talk between ER and IGF-IR on cellular proliferation, we used NWTB3 cells, which overexpress IGF-IR but are devoid of ER. These cells were transfected with ERα cDNA or vector control for 24 h and subsequently stimulated with 10 nM IGF-I for 48–72 h. In the presence of ER, cell proliferation was significantly increased after 48 and 72 h, respectively, as compared to cells without ER cDNA transfection. *N* = 3, *, *P*<0.05 as compare to cells without IGF-I treatment; #, *P*<0.05 as compare to the ERα-negative control groups.

To investigate the potential cross-talk between ERα and IGF-IR on cellular proliferation, we used NWTB3 cells, which overexpress IGF-IR but are devoid of ER. These cells were transfected with ERα cDNA or vector control for 24 h and subsequently stimulated with 10 nM IGF-I for 48–72 h. In the presence of ERα, cell proliferation was significantly increased after 48 h and 72 h as compared with cells without transfected with ERα cDNA (*P*<0.05, Figure 5). Therefore, ER expression had positive effects on cellular proliferation by IGF-I.

### ER distribution changes in intracellular of NWTB3 cells by ER transfection after IGF-I stimulation

**Figure 6:**
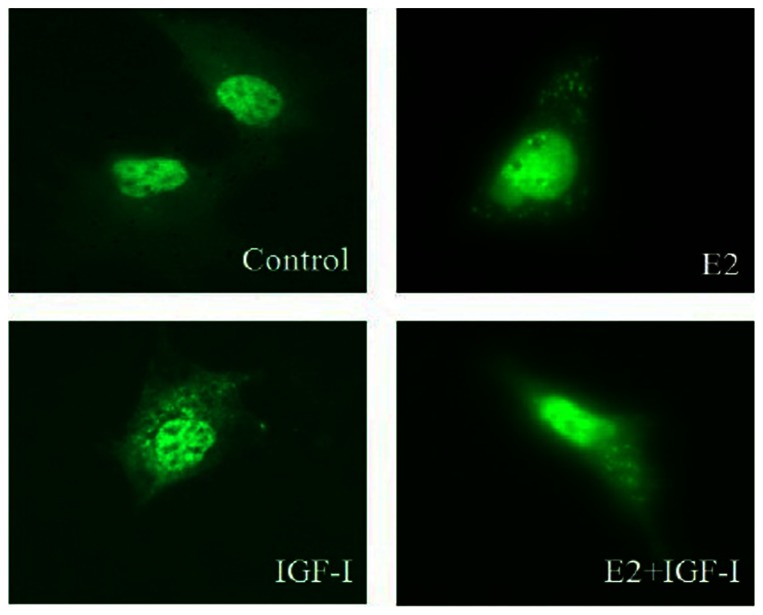
E2 and IGF-I quickly stimulate GFP-ERα translocation from nucleus to cytoplasm in NWTB3 cells. ER was mostly located in the nucleus in the non-treated control group, however, only a small part in the cytoplasm after E2 treatment (20 min). ER was significantly increased in the cytoplasm in IGF-I and IGF-I plus ER treatment groups (20 min).

NWTB3 cells were transfected with ERα cDNA for 24 hours, then stimulated with 10 nM E2, 10 nM IGF-I, or both at the same time. ER was mostly located in the nucleus in the non-treated control group and E2 treatment group, only a small part was in the cytoplasm (Figure 6). ER was significantly increased in the cytoplasm in IGF-I and IGF-I plus E2 treatment groups (Figure 6).

## Discussion

The additive or synergistic effects of IGF-I and E2 on cell proliferation [9], tumor development [23], anti-apoptosis [24], vascular protection [25] and neuroprotective effect [26] have been described. It has been demonstrated that E2 potentiated the IGF-I effect on IGF-IR signaling as well as cell cycle components [10]. On the other hand, our present study showed IGF-I and its receptor affected the association of IGF-IR with ERα, and ERα is involved in IGF-IR signaling pathway induced by IGF-I.

We found the association of IGF-IR with ERα was enhanced rapidly after IGF-I or E2 treatment in NWTB3 cells overexpressing IGF-IR and transiently transfected with an exogenous ERα expression plasmid. The association of IGF-IR with ERα was induced by IGF-I in MCF-7 cells as well. Thus, the association has been shown in wild type, epithelial and fibroblast derived cell lines. Furthermore, to confirm the function of IGF-IR in this association, we utilized MCF-7^SX13^ cells in which IGF-IR was reduced by 50% when compared to MCF-7 cells. IGF-I fails to enhance the association of IGF-IR to ERα in this cell. A previous study demonstrated that E2 potentiated the association ERα and IGF-IRs in COS7 and HEK293 cells [22]. E2 stimulated a putative membrane-associated binding site, which could be a form of ER or a membrane receptor, and induce a rapid intracellular signal transduction and tissue response. Not only could E2 potentiate the rapid association of ERα with IGF-IR and IGF-I signaling pathway [22], but also IGF-I could increase binding IGF-IR to ERα and probably improve biological function of IGF-I and E2. Our study extended the understanding of synergistic effect of E2 and IGF-I and nongenomic effect of E2. To our knowledge this is the first report of IGF-I inducing binding of IGF-IR to ERα.

It has recently been demonstrated that loss of ERα in MCF-7 cells was associated with reduced expression of critical IGF-signaling components (IGF-IR and IRS-1) and that this was associated with an inhibitory response to IGF-I [5]. These results suggest that ERα is a critical regulator of IGF-I signaling and growth in mammalian tumor cells. In agreement with previous results, we have shown here that introduction of ERα into NWTB3 cells increased basal level and rapidity of phosphorylation of IGF-I-signaling pathway downstream components (Erk1/2 and Akt). ERα transfected NWTB3 cells increased cell proliferation compared with ERα-negative control cells in the presence of stimuli (IGF-I). It was previously reported that ERα was activated by Ras-MAPK cascade of the growth factor signaling pathways [17]. Activated ERα rapidly bound to IGF-IR and induced phosphorylation of Akt and Erk1/2. Thus, IGF-I signaling was enlarged through ERα feedback.

In conclusion, we have demonstrated IGF-I induced association of IGF-IR and ERα. ERα and its association with IGF-IR potentiated IGF-I signaling pathways and cell growth. Although targeting ER has been one of the most successful approaches in cancer therapy, such as breast cancer, combined treatment with an IGF-IR tyrosine kinase inhibitor (NVPAEW541) has been shown to exhibit synergism in inhibiting proliferation and inducing apoptosis in breast cancer cells [27]–[28]. The cross-talk between ER and IGF-IR and its regulated molecular mechanisms observed in the present study provide further evidence for the potential clinical usefulness of targeting both ER and IGF-IR. Future animal studies and human clinical trials are needed to strategize the disruption of ER and IGF-IR interactions for breast cancer treatment.
